# Supplementary material for: Modeling glioblastoma heterogeneity as a dynamic network of cell states
Source: Mol Syst Biol. 2021 Sep 16;17(9):e10105. doi: 10.15252/msb.202010105 (PMC8444284; doi:10.15252/msb.202010105)
Supplement: Supplementary file 5 — Source Data for Figure 3 [file MSB-17-e10105-s001.zip › Figure3A_sourcedata/GSEA_3065/nosigdb_state6.GseaPreranked.1622718443993/RICHARDS_NEFTEL_CELL_2019_NPC1.html]

Details for gene set RICHARDS\_NEFTEL\_CELL\_2019\_NPC1[GSEA]

|  || Dataset | state6 |
| Phenotype | NoPhenotypeAvailable |
| Upregulated in class | na\_pos |
| GeneSet | RICHARDS\_NEFTEL\_CELL\_2019\_NPC1 |
| Enrichment Score (ES) | 0.68681043 |
| Normalized Enrichment Score (NES) | 2.3534193 |
| Nominal p-value | 0.0 |
| FDR q-value | 0.0 |
| FWER p-Value | 0.0 |
Table: GSEA Results Summary

  

Fig 1: Enrichment plot: RICHARDS\_NEFTEL\_CELL\_2019\_NPC1      
 Profile of the Running ES Score & Positions of GeneSet Members on the Rank Ordered List

  

| PROBE | GENE SYMBOL | GENE\_TITLE | RANK IN GENE LIST | RANK METRIC SCORE | RUNNING ES | CORE ENRICHMENT || 1 | SOX4 |  |  | 3 | 0.375 | 0.0976 | Yes |
| 2 | PCBP4 |  |  | 7 | 0.329 | 0.1830 | Yes |
| 3 | HES6 |  |  | 44 | 0.248 | 0.2436 | Yes |
| 4 | DLL3 |  |  | 49 | 0.246 | 0.3074 | Yes |
| 5 | BTG2 |  |  | 63 | 0.234 | 0.3670 | Yes |
| 6 | SOX11 |  |  | 70 | 0.230 | 0.4265 | Yes |
| 7 | GLCCI1 |  |  | 101 | 0.213 | 0.4786 | Yes |
| 8 | OLIG1 |  |  | 138 | 0.197 | 0.5259 | Yes |
| 9 | ETV1 |  |  | 196 | 0.177 | 0.5657 | Yes |
| 10 | MARCKSL1 |  |  | 223 | 0.169 | 0.6067 | Yes |
| 11 | FXYD6 |  |  | 387 | 0.138 | 0.6241 | Yes |
| 12 | CD24 |  |  | 674 | 0.108 | 0.6197 | Yes |
| 13 | SEZ6L |  |  | 733 | 0.103 | 0.6399 | Yes |
| 14 | AMOTL2 |  |  | 904 | 0.091 | 0.6442 | Yes |
| 15 | MAP2 |  |  | 917 | 0.090 | 0.6664 | Yes |
| 16 | PFN2 |  |  | 942 | 0.089 | 0.6868 | Yes |
| 17 | BEX1 |  |  | 1352 | 0.069 | 0.6582 | No |
| 18 | ABAT |  |  | 1484 | 0.065 | 0.6601 | No |
| 19 | NXPH1 |  |  | 1558 | 0.062 | 0.6679 | No |
| 20 | STMN1 |  |  | 1649 | 0.058 | 0.6729 | No |
| 21 | HIP1 |  |  | 1868 | 0.052 | 0.6614 | No |
| 22 | MEST |  |  | 1882 | 0.051 | 0.6733 | No |
| 23 | CHD7 |  |  | 1953 | 0.049 | 0.6781 | No |
| 24 | TUBA1A |  |  | 2135 | 0.044 | 0.6688 | No |
| 25 | MLLT11 |  |  | 2335 | 0.038 | 0.6559 | No |
| 26 | LRRN1 |  |  | 2640 | 0.030 | 0.6291 | No |
| 27 | TSPAN13 |  |  | 3733 | 0.006 | 0.5061 | No |
| 28 | TCF12 |  |  | 5481 | -0.028 | 0.3140 | No |
| 29 | TAGLN3 |  |  | 5582 | -0.030 | 0.3105 | No |
| 30 | DBN1 |  |  | 5587 | -0.030 | 0.3179 | No |
| 31 | SERINC5 |  |  | 5716 | -0.033 | 0.3119 | No |
| 32 | LMF1 |  |  | 6150 | -0.041 | 0.2733 | No |
| 33 | PTPRS |  |  | 6439 | -0.048 | 0.2530 | No |
| 34 | BCAN |  |  | 6866 | -0.061 | 0.2202 | No |
Table: GSEA details [plain text format]

  

Fig 2: RICHARDS\_NEFTEL\_CELL\_2019\_NPC1: Random ES distribution      
 Gene set null distribution of ES for **RICHARDS\_NEFTEL\_CELL\_2019\_NPC1**
